# Feasibility of Diffusion Tensor Imaging for Decreasing Biopsy Rates in Breast Imaging: Interim Analysis of a Prospective Study

**DOI:** 10.3390/diagnostics13132226

**Published:** 2023-06-30

**Authors:** Jacob S. Ecanow, David B. Ecanow, Bradley Hack, Nondas Leloudas, Pottumarthi V. Prasad

**Affiliations:** Department of Radiology, NorthShore University HealthSystem, Evanston, IL 60201, USApprasad@northshore.org (P.V.P.)

**Keywords:** breast cancer, magnetic resonance imaging, diffusion tensor imaging, biopsy

## Abstract

Because of the limited specificity of diagnostic imaging, many breast lesions referred for biopsy turn out to be benign. The objective of this study was to evaluate whether diffusion tensor MRI (DTI) parametric maps can be used to safely avoid biopsy of breast lesions. Individuals referred for breast biopsy based on mammogram (MG), ultrasound (US), and/or contrast enhanced (CE)-MRI were recruited. Scans consisting of T2-weighted and DTI sequences were performed. Multiple DTI-derived parametric color maps were evaluated semi-quantitatively to characterize lesions as “definitely benign,” “not definitely benign,” or “suspicious.” All patients subsequently underwent biopsy. In this moderately-sized prospective study, 21 out of 47 pathologically proven benign lesions were characterized by both readers as “definitely benign,” which would have precluded the need for biopsy. Biopsy was recommended for 11 out of 13 cancers that were characterized as “suspicious.” In the remaining two cancers and 26 of 47 benign lesions, the scans were characterized as “not definitely benign” and hence required biopsy. The main causes for “not definitely benign” scans were small lesion sizes and noise. The results suggest that in appropriately selected patients, DTI may be used to safely reduce the number of unnecessary breast biopsies.

## 1. Introduction

Adenocarcinoma of the breast is the leading cause of cancer death in women worldwide [1]. The early diagnosis of breast cancer through screening mammography (MG) has been proven to significantly reduce mortality but is subject to significant limitations including modest sensitivity, which gives rise to a 30–50% interval cancer rate, as well as a low positive predictive value (PPV), leading to a large burden of follow-up and many breast biopsies [2,3,4]. Dense breast tissue, which is encountered in over 50% of women, further decreases the sensitivity and specificity of mammography [2]. Ultrasound (US) is used for both diagnosis and supplemental screening but is also associated with a low PPV and leads to further increased cost and additional biopsy recommendations [2,5]. Some reports indicate that in current practice, up to 80% of breast lesions that undergo biopsy turn out to be benign [6,7]. As a result, the number of breast biopsies has skyrocketed, with some estimates suggesting that the annual rate of these procedures in the United States alone may be as high as 1,000,000 per year [7]. This places a high economic burden on society, estimated to be USD 2 billion annually [8], and is emotionally burdensome to patients [9].

The use of breast MRI as a non-invasive test to exclude malignancy and preclude an otherwise unnecessary breast biopsy is appealing. Breast MRI currently has higher sensitivity (>90%) than MG or US and is routinely used for screening in high-risk populations as well as for the loco-regional staging of breast cancer [3]. In order to achieve high sensitivity, current breast MRI protocols require the use of intravenous (IV) gadolinium-based contrast agents (GBCAs). However, dynamic contrast-enhanced MRI (DCE-MRI) has several significant limitations that make it suboptimal for many diagnostic indications, including a high false positive rate, high cost, the need for IV cannulation, and potential side effects of the GBCA [6,10]. Up to one-third of women undergoing DCE-MRI may be called back for follow-up or biopsy, and up to 40% of the resultant breast biopsies are benign [11,12]. Consequently, similar to the situation with MG and US, DCE-MRI scans frequently lead to additional biopsies for lesions that appear suspicious but are subsequently evaluated as benign [5].

Diffusion-weighted imaging (DWI) has been shown to reduce the number of unnecessary biopsies indicated by DCE-MRI [10]. Much of the current literature on DWI uses quantitative analysis to characterize lesions, and a threshold value of 1.5 × 10^−3^ mm^2^/s for the apparent diffusion coefficient (ADC) is widely accepted as a value below which a lesion is considered to be suspicious for malignancy [10,13,14,15]. A variant of DWI is diffusion tensor imaging (DTI), which can characterize the directional changes in diffusion coefficients [16]. DTI affords multiple quantitative parameters, including the highest principal diffusion coefficient (λ1), maximal anisotropy (λ1–λ3), and mean diffusivity, also termed the apparent diffusion coefficient (MD/ADC), all of which have the potential to better characterize breast lesions [17,18]. Of note, given that the λ1 is higher in magnitude than the ADC, the threshold value for this parameter is slightly higher, 1.7 × 10^−3^ mm^2^/s. Additionally, DTI data can be displayed in the form of color-coded parametric maps that can help highlight breast lesions [17,19,20,21]. To date, studies have suggested that DTI may become a successful strategy for improving the specificity of breast MRI [22,23,24].

The present study was designed as a prospective trial to determine how DTI might be used in routine clinical practice in order to potentially reduce the number of unnecessary breast biopsies. To date, the studies that evaluated the potential role of DWI in reducing biopsies were performed in individuals referred for clinical breast MRI [10,15]. As articulated earlier, breast MRI is reserved for a small subset of patients and so in current practice, most biopsy recommendations arise from MG and US examinations that do not require a follow-up breast MRI. The aim of this study was to apply this emerging MRI breast application primarily to all these cases, whether or not a follow-up breast MRI was indicated.

Additionally, while a quantitative regions of interest (ROIs) analysis is a strong tool for clinical research, it is somewhat cumbersome in a routine clinical environment in which the average time spent per case is typically less than 10 min. Consequently, the other aim was to evaluate the feasibility of using a semi-quantitative visual approach in which color maps of the three DTI indices would be used to triage the need for biopsy. For such an approach, the λ1, MD, and λ1–λ3 color maps were set by choice of color bars using previously determined threshold values to detect malignant lesions [16,17,18,20]. The specific goal of this study was to determine whether a rapid non-contrast-enhanced MRI examination consisting of “anatomic” T2 sequences and DTI images acquired prior to biopsies of indeterminate breast lesions and presented as color maps can reliably identify benign lesions that do not require biopsy without reducing the cancer detection rate.

## 2. Materials and Methods

### 2.1. Subjects and Study Design

**Figure 1:**
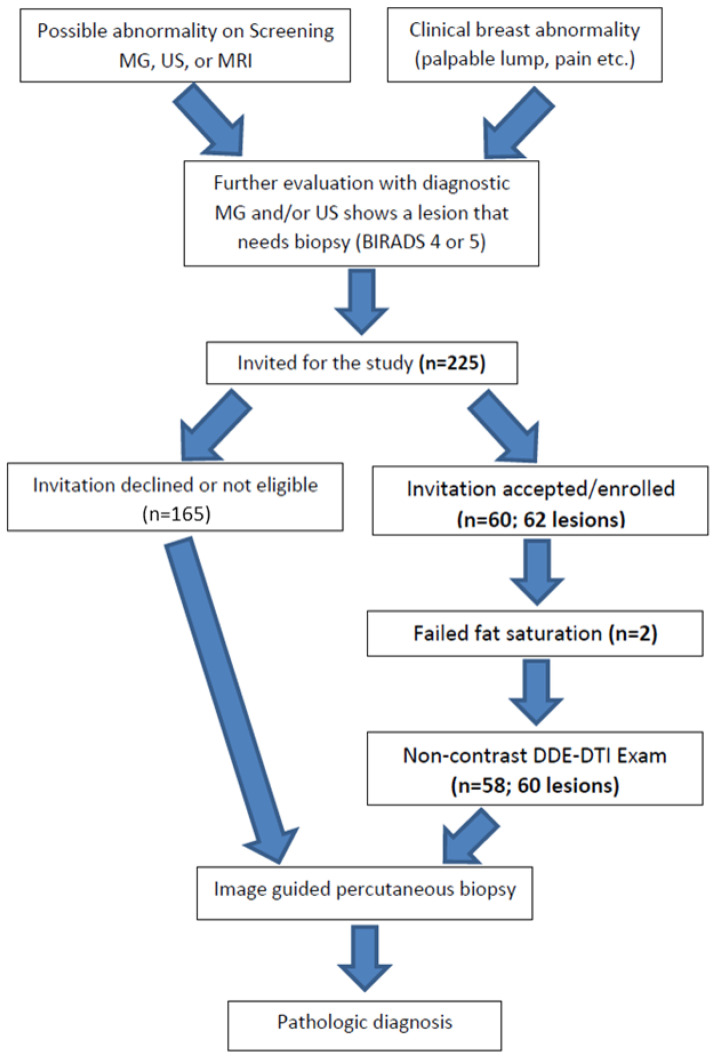
Study pathway: describes the way the participants were enrolled in the study and the flow of events during and following the study procedures.

The protocol for this HIPPA-compliant prospective study was approved by the institutional review board. Signed informed consent was obtained from all participants. Female patients aged 18 years or older with at least one newly detected BIRADS 3, 4, or 5 breast lesion who were scheduled to undergo an image-guided biopsy were eligible to participate (see Figure 1). Patients were ineligible if they had any contraindication to MRI scanning or if they were unable to provide informed consent. All patients had previously undergone a MG, US, or DCE-MRI of the breasts or had been imaged with a combination of modalities. Breast biopsies were performed under US, stereotactic, or MRI guidance as part of the patient’s clinical breast care. Demographic data were recorded (Table 1). The mammographic, ultrasound, and DCE-MRI findings, sizes, and assessments for each lesion, as well as the breast density assessments for each subject, were abstracted from the clinical radiology reports and recorded using the standard BI-RADS nomenclature [25].

### 2.2. MRI Acquisition

All breast MRI scans were performed using a 3T Siemens Skyra^Fit^ whole body scanner (Siemens Healthcare, Erlangen, Germany) with a dedicated 8-channel breast coil (Sentinelle, Montréal, QC, Canada). Single-shot, echo-planar diffusion-weighted images with fat saturation were acquired in the axial plane with two b-values (0 and 700 s/mm^2^) applied along 30 different directions and an echo time = 90 ms. Images were obtained with 2 mm isotropic resolution. Dynamic field correction was applied with a total acquisition time of ~8 min. Axial non-fat-suppressed turbo spin echo (TSE) T2-weighted and T2 TIRM images were obtained; the T2-weighted images were obtained with the same slice thickness as the DTI to facilitate correlation (See Table 2 with parameters).

### 2.3. Image Processing

The DICOM data were transferred to a dedicated secure workstation for post processing using BIT-Motion software (DDE MRI Solutions Inc., Tel Aviv, Israel), which yielded the 3 eigenvectors (v1, v2, and v3) defining the principal diffusion directions in the orthogonal axes of the breast tissue, as well as the corresponding eigenvalues (λ1, λ2, λ3) which quantified the magnitude of the principal diffusion coefficient for each pixel, sorted from highest to lowest [16,20]. MD or ADC [(λ1 + λ3 + λ1)/3], maximal anisotropy (λ1–λ3), and fractional anisotropy (FA) were calculated from the three diffusion coefficients. The data were displayed as color-coded parametric maps that were co-registered with the B0 and optionally T2-weighted axial images.

### 2.4. Data Analysis

**Figure 2:**
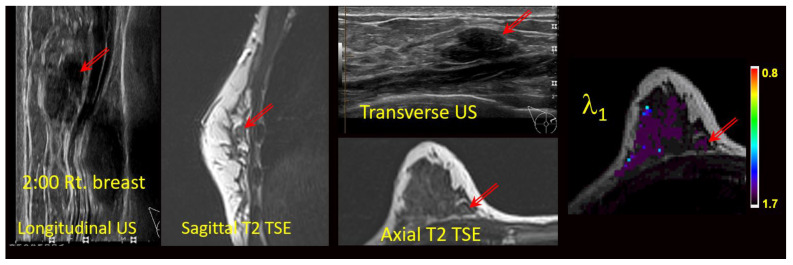
Anatomic correlation with prior multimodal clinical images and current anatomical T2-weighted images (axial T2 TSE) with DTI-derived axial λ1 parametric map overlaid on a the T2 weighted image. Units for l1 are 10^−3^ mm^2^/s. Arrows indicate the lesion.

**Figure 3:**
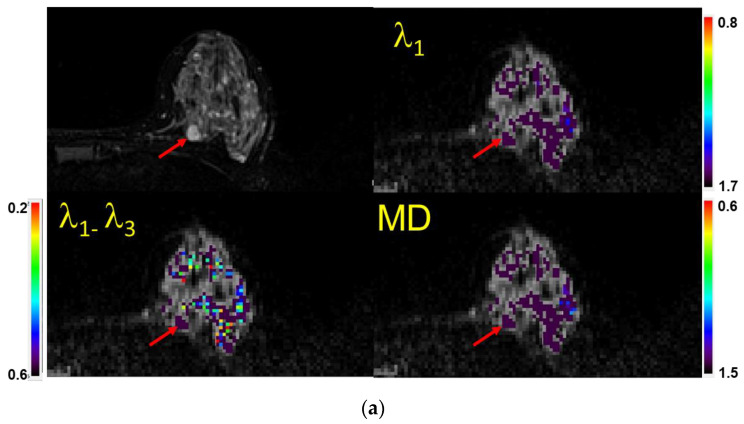

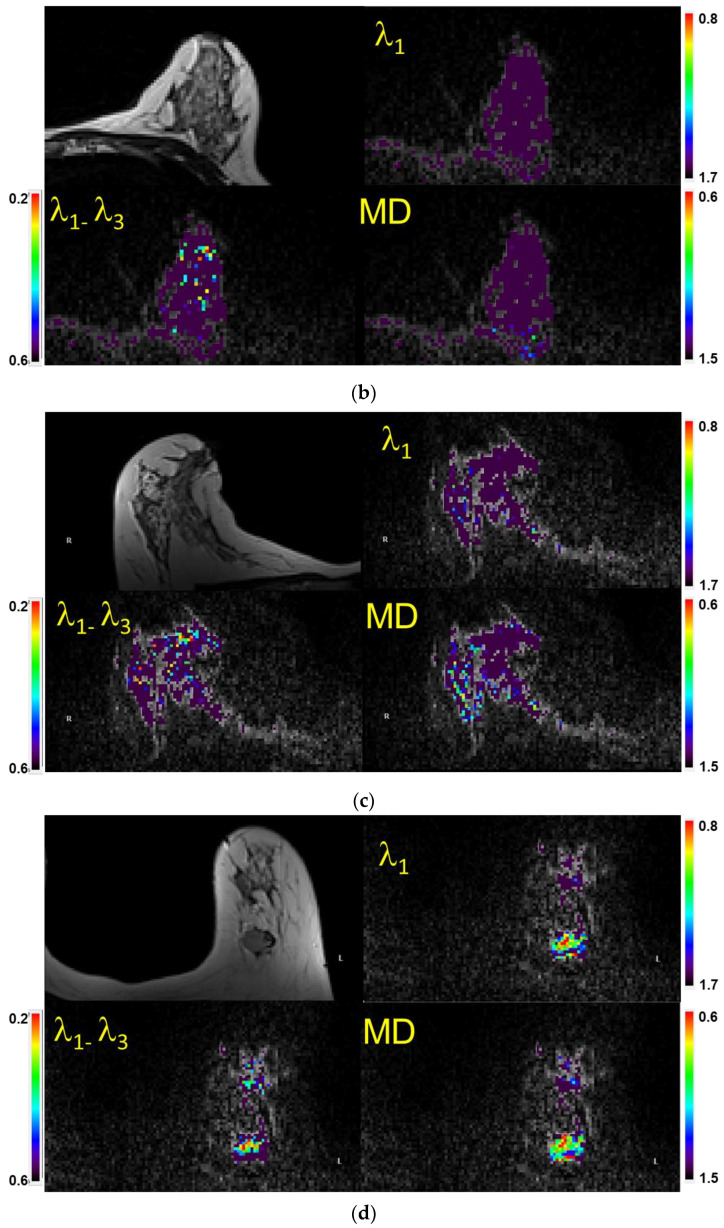
(**a**) Example of a case with a lesion (mass) identified (visual score = 2) on a fatsat T2-weighted MRI (top left), characterized as benign semi-quantitatively, and confirmed quantitatively. Shown are the λ1 map, MD/ADC map, and maximal anisotropy (λ1–λ3) map. Note that the color pixels in the mass are uniformly purple on the λ1 and MD maps, confirming that the lesion is benign (Bx score = 0). Recommendation: No biopsy. Subsequent biopsy confirmed a benign fibroadenoma. The values of λ1, MD, and λ1–λ3 in the corresponding scales are ×10^−3^ mm^2^/s. Arrows indicate the lesion. (**b**) Example of a case with no lesion identified on MRI (visual score = 0) and in which=ch a biopsy was not indicated. When no lesion could be identified, the readers used the fact that there were no visible non-purple pixels within the breast parenchyma in the expected region of the lesion to score the case as benign (Bx score = 0). Shown are the T2-weighted image (Top left), λ1 map, MD/ADC map, and maximal anisotropy (λ1–λ3) map overlaid on the corresponding B0 image. Recommendation: No biopsy. The values of λ1, MD, and λ1–λ3 in the corresponding scales are ×10^−3^ mm^2^/s. (**c**) Example of a case with no lesion identified on MRI (visual score = 0). When no lesion could be identified and there were multiple non-purple pixels within the breast parenchyma in the area of interest, the scan was scored as indeterminate (Bx score = 1). Recommendation: Proceed with biopsy. Shown are atheT2-weighted image (Top left), λ1 map, MD/ADC map, and (λ1–λ3) map overlaid on the corresponding B0 image. The random distribution of non-purple pixels is a result of limited signal to noise in the diffusion-weighted images. The values of λ1, MD, and λ1–λ3 in the corresponding scales are ×10^−3^ mm^2^/s. (**d**) Example of a case with a mass identified (vis. score = 2) on a fatsat T2-weighted MRI (top left) and characterized as malignant both semi-quantitatively and quantitatively (Bx score = 2). Shown are the λ1 map, MD/ADC map, and anisotropy (λ1–λ3) map overlaid on the corresponding B0 image. Note the non-purple color pixels in the mass. Recommendation: Biopsy. Subsequent biopsy confirmed an invasive ductal carcinoma (IDC). The values of λ1, MD, and λ1–λ3 in the corresponding scales are ×10^−3^ mm^2^/s.

The MG, US, and/or DCE-MRI images from prior clinical scans for each lesion were correlated with the study-related T2-weighted TSE and/or TIRM images and the DTI maps (Figure 2) by two board certified subspecialty breast imaging radiologists, each with more than 10 years of experience, who were unaware of the final pathology. The visibilities of the lesions on the DTI studies were recorded using a 3-point scale (0 = not visible; 1 = questionably visible; 2 = definitely visible and able to characterize) (Table 3 and Figure 3).

The color bars were set for each of the three DTI measures as follows: λ1: (0.8 − 1.7) × 10^−3^ mm^2^/s, MD: (0.6 − 1.5) × 10^−3^ mm^2^/s, and λ1–λ3: (0.2 − 0.6) × 10^−3^ mm^2^/s. Pixel values above the highest value are displayed in purple, while all values below the lowest value in the range appear red. A homogenous purple color indicates benign parenchyma. Basing their interpretations primarily on the λ1 and MD maps, the two radiologists scored each exam on a three-point scale: 0 = no biopsy recommended (benign); 1= biopsy (indeterminate); or 2 = biopsy (suspicious). Maximum anisotropy maps usually had multiple non-purple pixels and therefore were not used for interpretation but were included in the quantitative analysis in visible masses. Figure 3 illustrates how the scoring system was applied to each lesion depending on whether it was specifically visualized on the non-CE MRI or not.

In order to validate the interpretations, for all cases in which the lesions were coded as specifically identified (visual score of 2), ROIs were placed by one of the Radiologists on the lesion and on the corresponding normal parenchyma of the breast as a control.

### 2.5. Statistical Analysis

The inter-reader agreement was assessed using an intra-class-coefficient. A value above 0.9 was considered excellent, 0.8 < ICC < 0.89 was considered good, and 0.8 < ICC < 0.79 was considered acceptable. To compare the quantitative region of interest measurements between the three groups (control, benign, and malignant), a non-parametric one-way ANOVA (Kruskal–Wallis) was used. *p* < 0.05 was considered statistically significant. 

All analyses were performed using SPSS 22.0 software (IBM Corp., Armonk, NY, USA).

## 3. Results

To date, 225 subjects were invited to participate; 165 declined or were not eligible, and 60 (age 50.3 ± 13.4) were enrolled and underwent a non-contrast DTI exam prior to undergoing their breast biopsy. The DTI scans of two patients, one with IDC and one benign, were technical failures due to sub-optimal fat saturation. Two patients had two lesions, for a total of 60 lesions whose scans could be evaluated to determine whether biopsy could be avoided, which formed the basis of this report. The types of lesions, both benign and malignant, are listed in Table 1.

### 3.1. Semi-Quantitative Evaluation of the DTI Scans 

Table 4 summarizes the two readers’ classifications in terms of lesion visibility on MRI and whether they felt confident that a biopsy was not indicated.

Overall, there was an excellent agreement between the two readers, with ICC > 0.9 for both lesion visibility (ICC = 0.953) and whether biopsy was indicated (ICC = 0.952). Reader 1 was relatively more conservative, and the differences between the readers were seen only at scores 0 and 2.

### 3.2. Quantitative Evaluation of the Visible Lesions

**Figure 4:**
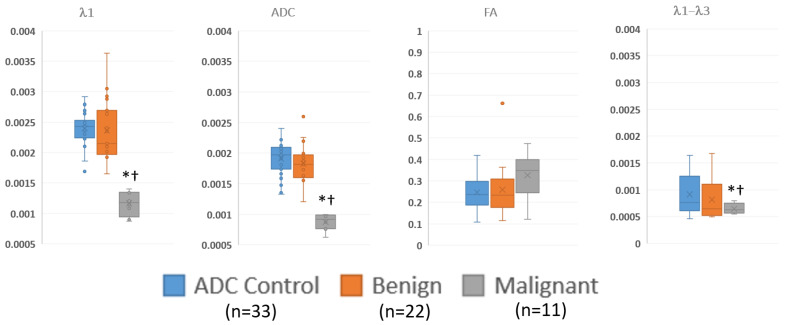
Summary of ROI measurements of diffusion tensor measurements in visible benign, and malignant lesions and control regions away from the lesions via box and whisker plot. λ1, MD/ADC, and λ1–λ3 have units of mm^2^/s. * *p* < 0.05 compared to control; † *p* < 0.05 compared to benign.

Figure 4 and Table 5 summarize the quantitative regions of interest data. Malignant lesions had distinctly lower values in both λ1 and MD and were consistent with prior threshold values. While maximum diffusivity, λ1–λ3, had overlapping values, they were deemed statistically lower in malignant lesions. On the other hand, FA was not different between groups.

## 4. Discussion

To our knowledge, this is the first prospective study evaluating the performance of a standalone non-contrast DTI exam to categorize indeterminate breast lesions as either definitely benign (and therefore precluding a recommended biopsy) or alternatively, “not definitely benign”, using histopathology as the gold standard. The lesions in the “not definitely benign” category were either indeterminate (recommendation to proceed with biopsy based on the prior imaging recommendation) or “suspicious” (confirming the biopsy recommendation). This study was built on prior reports that confirmed the accuracy of DTI, but instead of focusing on quantitative measurements of the directional diffusion coefficients in visible lesions, the primary approach was centered on the semi-quantitative visual evaluation of color-coded λ1 and MD/ADC maps, which is a practical method that could be used in routine clinical practice. Using this approach, reader 1 was able to safely reduce the biopsy rate for benign lesions by 45% (21/47) and the overall biopsy rate by 33% (21/60), and reader 2 avoided 51% of unnecessary biopsies (24/47) with an overall reduction rate of 40% (24/60). While most of the patients had been imaged by MG or US, these results are similar to published results using DWI to reduce the biopsy rate for lesions seen on DCE-MRI. Clauser et al. showed a 32.6% reduction in the biopsy rate when DWI was added to DCE-MRI, using an ADC threshold of 1.5 × 10^−3^ mm^2^/s [10].

The study population included 23% (14/61) biopsy-proven malignancies, which is consistent with the “positive biopsy rate” for general breast imaging that is reported in the literature (~20%) [7]. None of these cancers were classified as “definitely benign”. While DTI color maps could not be generated in one of the cancer patients, in the remaining 13 biopsy-proven cancers, DTI was able to correctly characterize 11 lesions as specifically suspicious. The scans of the other two cancers were “not definitely benign”, and biopsy would not have been precluded. One of these two cancers was a 3 mm invasive lobular carcinoma that was below the spatial resolution of DTI, and the scan of the other had non-purple pixels in the area of the lesion.

Without the benefit of GBCA enhancement to localize lesions, the strategy relied on either the identification of “hot spots” on the DTI color maps (Figure 3d) or else careful correlation of the “anatomic” T2 sequences with the original imaging and with the DTI scans (Figure 2). Anatomic correlation with MG and US included matching the shape of the lesion and the appearance of the local anatomy as well as careful localization with respect to the nipple and chest wall. Prior studies have addressed the visibility of lesions on diffusion-weighted imaging without contrast and reported that malignant masses larger than 5–10 mm could be demonstrated on DWI without DCE and that DWI-guided biopsies were therefore feasible in these lesions [26,27]. Montemezzi et al. reported that 75/87 lesions larger than 5 mm were visible on DWI [27]. Sixty-four of the lesions underwent successful biopsy under DWI guidance alone.

In this study, reader 1 rated three malignant lesions as visible by virtue of the fact that they had red pixels (hot spots) in an area with otherwise no signal or uniform purple signal. The other eight cancers were visible on the T2 or TIRM images and also had red pixels inside. For the second reader, one cancer was identified using the “Hot-spot” method, and the others were rated as definitely visible by correlating the T2 and DTI studies with the original imaging.

The visibility of a malignant mass as a “hot spot” on DTI also depends on histology and MRI morphology. For example, diffusion imaging is less sensitive to lobular cancers, which tend to have infiltrative histologic patterns [28]. While Berger et al. were able to see five out of six IDC masses, they found that only one of three ILC lesions, and they reported that none of their DCIS lesions were clearly visible on DWI [26]. In the current study, the 3 mm mass that was undetectable was an ILC, which likely also contributed to the lack of visualization. DCIS lesions, with their similar non-mass-like histologies and corresponding non-mass-like enhancement patterns on imaging, are also less detectable on DWI/DTI, and this may have contributed to a second cancer that could not be seen [29]. These results suggest that the use of DTI for the triage of indeterminate breast lesions that have already been characterized by MG, US, or DCE-MRI may be limited to mass-forming lesions larger than 7 mm, unless further improvements in spatial resolution are feasible.

DTI data were acquired at 2 mm isotropic resolution at 3T; however, the signal-to-noise ratio (SNR) was found to be limited, especially in fatty breasts. The one cancer that was missed due to poor fat saturation was the only individual with a BIRADS breast density A. The proposed semi-quantitative evaluation would improve with a higher SNR so as to limit the non-purple pixels. With this improvement, it is possible to envision a non-contrast MRI examination as an option to offer individuals referred for a biopsy. SNR improvements may require better RF coils to accommodate different breast sizes and afford accelerated acquisitions such as simultaneous multi-slice (SMS) [30]. Such coils combined with segmented EPI acquisitions should allow for a much-improved image quality with a higher SNR [30,31]. The proposed semi-quantitative approach would obviate the need for quantitative ROI measurements and would be more clinically practical in terms of throughput.

The quantitative analysis, which was performed to verify the threshold values used, was consistent with prior reports and supported the use of DTI parameters (λ1, λ1–λ3, and MD/ADC) but not normalized measures (such as FA) in discriminating benign lesions from malignancies [19]. ROI measurements were performed on all visible lesions in order to validate the semi-quantitative judgements on whether (a) the lesion was visible and (b) likely benign, indeterminate, or suspicious. There was a statistically significant separation between malignant lesions and both benign lesions and background parenchyma on the λ1, MD, and λ1–λ3 maps when using the threshold values that were established in the literature. This supports the accuracy of the visual assessment of the color maps.

The study had several limitations. All patients in our clinic undergo a consultation with a radiologist when a biopsy is recommended. The subjects were recruited after this consultation, and there was likely a selection bias towards less aggressive lesions because the patients with more suspicious lesions may have experienced heightened anxiety after the consultation and were therefore less inclined to participate. However, since the interest was to primarily identify “definitely benign” lesions, this was not a critical limitation. There may also have been an observer bias as the radiologists reviewed the DTI data in conjunction with prior imaging data. However, this is how the scans would be used in clinical practice, and therefore, the same conditions would apply. The relatively small number of participants may have limited the opportunity to observe more false negatives. Finally, the necessity of using only non-CE MRI meant that in some cases, the true location of the lesion could not be determined, and therefore true analyses of these lesions were not possible. However, as we were interpreting the DTI scan as a “Hot-spot” scan with the purpose of deciding “biopsy” or “no biopsy”, this limitation did not invalidate the conclusions.

## 5. Conclusions

The data presented suggest that DTI could be used to reduce the number of unnecessary biopsies that arise in clinical breast imaging as it is currently practiced. The proposed semi-quantitative analysis method may also be amenable to the use of artificial intelligence and deep learning approaches in the future. The performance of this approach could be improved with improved spatial resolution and increased SNR, which necessitate better RF coils and novel sequences such as segmented EPI. Performance could also benefit from the stronger gradient systems that are on the horizon. While the lack of DCE MRI limited the visualization of masses, the specific identification a mass is only necessary if a quantitative ROI analysis is to be applied for every lesion, which is not practical in routine clinical practice. Visualization is also important in raising the confidence of the interpreting physician in making a conclusion about the diagnosis, but the proposed approach to reading the scan as a hot spot scan offsets that necessity to some extent. This preliminary experience supports the use of semi-quantitative reads; however, to make them robust for routine clinical use, it is necessary to improve the SNR. A cost–benefit analysis may also be necessary prior to translating DWI/DTI into routine clinical practice; however, an important factor that should be taken into account in such an analysis is the human cost in terms of the anxiety that is generated by a recommendation for breast biopsy.

## Figures and Tables

**Table 1:** Demographic data for participants, summarizing the relevant patient and lesion information. The majority of participants had heterogeneously dense breasts with a BI-RADS score of 4 and were recommended for biopsy based on mammograms. The majority of lesions were larger than 1 cm.

**Participants Consented (*n* = 62)**		
**Age**		
50.9 ± 13.6	***n***	**%**
**Breast density (*n* = 62)**		
Unknown	4	6.5
(A) Fatty	1	1.6
(B) Scattered fibroglandular tissue	12	19.4
(C) Heterogeneously dense	38	61.3
(D) Extremely dense	7	11.3
**BI-RADS (*n* = 62)**		
3	7	11.7
4	47	73.3
5	8	13.3
**Original Presentation (*n* = 62)**		
Mammogram	41	66.1
ABUS	11	17.7
MRI	4	6.5
Palpable	6	9.7
**Histopathology (*n* = 61)**		
Benign	47	77.0
Malignant	14	23.0
Lesion size (cm)		
<1.0	17	27.9
1–1.9	30	49.2
≥2.0	8	13.1
Not apparent	6	9.8
**Lesions biopsied per patient (*n* = 61)**		
1	57	96.6
2	2	3.4
**Cancer Type (*n* = 14)**		
IDC	8	57.2
ILC	1	7.1
DCIS	2	14.3
Mixed	3	21.4
**Benign subtype (*n* = 47)**		
Normal breast tissue	7	14.9
Benign intramammary node	1	2.1
Fibroadenoma	18	38.3
Fat necrosis	2	4.3
Fibrocystic changes (FCD) *	9	19.1
PASH	6	12.8
Radial Scar/CSL	2	4.3
ALH	2	4.3
ADH	1	2.1

* Includes sclerosing adenosis, stromal fibrosis, and apocrine metaplasia.

**Table 2:** Key MRI acquisition parameters for T2-weighted and DTI sequences at 3T.

Parameter	T2-wtd TSE	Fat-Sat T2	DTI
**Sequence**	Turbo SE	Turbo IR Magnitude	Single Shot SE-EPI
**Field of View**	400 × 350 mm	370 × 403 mm	400 × 350 mm
**Slice Thickness**	2 mm	2 mm	2 mm
**Repetition Time**	2400 ms	5320 ms	9100 ms
**Echo Time**	83 ms	82 ms	90 ms
**Inversion Time**	n/a	220 ms	n/a
**B value**	n/a	n/a	0, 700 s/mm^2^
**No. of Diffusion directions**	n/a	n/a	30
**Average**	2	2	1
**GRAPPA**	2	2	2
**Flip angle**	120°	120°	90°
**Matrix**	256 × 192	320 × 278°	192 × 144
**# of Slices**	60	60	60
**Turbo factor**	9	10	n/a
**Bandwidth**	219 Hz/pixel	244 Hz/pixel	1370 Hz/pixel
**Slice Interval**	2 mm	2 mm	2 mm

**Table 3:** Lesion characterization scheme summarizing the criteria used to determine the interpretation of characterizing the lesions and the hypothetical recommendation for biopsy.

Feature	Interpretation	Recommendation
Lesion visible (2) and all purple.	Definitely benign (Bx = 0)	No biopsy (see Figure 3a)
Lesion not visible (0, 1), but there are only purple pixels in area of the lesion (no noise).	Definitely benign (Bx = 0)	No biopsy (see Figure 3b)
Lesion not visible (0, 1), but there are colored pixels or noise in the area.	NOT definitely benign (Indeterminate) (Bx = 1)	No change to the existing biopsy recommendation (see Figure 3c)
Lesion visible (2) and contains any colored pixels.	NOT definitely benign (Indeterminate) (Bx = 1)	No change to the existing biopsy recommendation
Lesion visible (2) and contains many colored pixels with red pixels.	Suspicious (Bx = 2)	Biopsy (see Figure 3d)

**Table 4:** Inter-reader agreement: summarizing the scoring of the lesions by the two readers and their recommendations for biopsy.

	Lesion Visibility		Biopsy/No-Biopsy	
Score	Reader 1	Reader 2	Reader 1	Reader 2
**0**	22	18	21	26
**1**	16	16	29	23
**2**	23	27	10	11

Visibility assessed using a three-point scale: 0 = not visible; 1: questionable visible; 2 = definitely visible. Biopy vs. no-biopsy was assessed on a three-point scale: 0 = definitely benign (no-biopsy); 1 = indeterminate (biopsy); 2 = suspicious (biopsy).

**Table 5:** Summary of regions of interest data, showing the numerical values of the data displayed in Figure 4. The malignant lesions have significantly lower λ1, ADC and λ1–λ3 values compared to either benign lesions or control regions.

		*n*	Mean	Std. Deviation
**λ1 mm^2^/s**	Control	33	2.35 × 10^−3^	2.63 × 10^−4^
	Benign	22	2.37 × 10^−3^	4.77 × 10^−4^
	Malignant	11	1.18 × 10^−3^	2.82 × 10^−4^
**ADC mm^2^/s**	Control	33	1.89 × 10^−3^ *^†^	2.66 × 10^−4^
	Benign	22	1.85 × 10^−3^	2.91 × 10^−4^
	Malignant	11	9.18 × 10^−4 *†^	2.47 × 10^−4^
**FA**	Control	32	0.235	0.098
	Benign	22	0.256	0.118
	Malignant	11	0.296	0.114
**λ1–λ3 mm^2^/s**	Control	33	8.92 × 10^−3^	4.61 × 10^−2^
	Benign	22	9.89 × 10^−4^	5.29 × 10^−4^
	Malignant	11	5.22 × 10^−4^ *^†^	1.90 × 10^−4^

* *p* < 0.05 compared to control; † *p* < 0.05 compared to benign via Kruskal–Wallis test.

## Data Availability

The data presented in this study are available upon request from the corresponding author. The data are not publicly available due to privacy concerns of the participants. Given the exploratory nature of the study, the consent form stipulated that the data not be shared outside of our institution.

## References

[1] Galati F., Moffa G., Pediconi F. (2022). Breast imaging: Beyond the detection. Eur. J. Radiol..

[2] Kuhl C.K., Strobel K., Bieling H., Leutner C., Schild H.H., Schrading S. (2017). Supplemental Breast MR Imaging Screening of Women with Average Risk of Breast Cancer. Radiology.

[3] Niell B.L., Freer P.E., Weinfurtner R.J., Arleo E.K., Drukteinis J.S. (2017). Screening for Breast Cancer. Radiol. Clin. N. Am..

[4] Qaseem A., Lin J.S., Mustafa R.A., Horwitch C.A., Wilt T.J., Clinical Guidelines Committee of the American College of Physicians (2019). Screening for Breast Cancer in Average-Risk Women: A Guidance Statement from the American College of Physicians. Ann. Intern. Med..

[5] Lehman C.D., Arao R.F., Sprague B.L., Lee J.M., Buist D.S., Kerlikowske K., Henderson L.M., Onega T., Tosteson A.N., Rauscher G.H. (2017). National Performance Benchmarks for Modern Screening Digital Mammography: Update from the Breast Cancer Surveillance Consortium. Radiology.

[6] Amornsiripanitch N., Bickelhaupt S., Shin H.J., Dang M., Rahbar H., Pinker K., Partridge S.C. (2019). Diffusion-weighted MRI for Unenhanced Breast Cancer Screening. Radiology.

[7] Johnson J.M., Johnson A.K., O’Meara E.S., Miglioretti D.L., Geller B.M., Hotaling E.N., Herschorn S.D. (2015). Breast cancer detection with short-interval follow-up compared with return to annual screening in patients with benign stereotactic or US-guided breast biopsy results. Radiology.

[8] Vlahiotis A., Griffin B., Stavros A.T., Margolis J. (2018). Analysis of utilization patterns and associated costs of the breast imaging and diagnostic procedures after screening mammography. Clin. Outcomes Res..

[9] Maimone S., Harper L.K., Mantia S.K., Advani P.P., Hochwald A.P., Li Z., Hines S.L., Patel B. (2023). MRI phenotypes associated with breast cancer predisposing genetic variants, a multisite review. Eur. J. Radiol..

[10] Clauser P., Krug B., Bickel H., Dietzel M., Pinker K., Neuhaus V.F., Marino M.A., Moschetta M., Troiano N., Helbich T.H. (2021). Diffusion-weighted Imaging Allows for Downgrading MR BI-RADS 4 Lesions in Contrast-enhanced MRI of the Breast to Avoid Unnecessary Biopsy. Clin. Cancer Res..

[11] Pinkney D.M., Chikarmane S.A., Giess C.S. (2019). Do benign-concordant breast MRI biopsy results require short interval follow-up imaging? Report of longitudinal study and review of the literature. Clin. Imaging.

[12] Spiegel T.N., Esplen M.J., Hill K.A., Wong J., Causer P.A., Warner E. (2011). Psychological impact of recall on women with BRCA mutations undergoing MRI surveillance. Breast.

[13] Baltzer A., Dietzel M., Kaiser C.G., Baltzer P.A. (2016). Combined reading of Contrast Enhanced and Diffusion Weighted Magnetic Resonance Imaging by using a simple sum score. Eur. Radiol..

[14] Baltzer P., Mann R.M., Iima M., Sigmund E.E., Clauser P., Gilbert F.J., Martincich L., Partridge S.C., Patterson A., Pinker K. (2020). Diffusion-weighted imaging of the breast-a consensus and mission statement from the EUSOBI International Breast Diffusion-Weighted Imaging working group. Eur. Radiol..

[15] Rahbar H., Zhang Z., Chenevert T.L., Romanoff J., Kitsch A.E., Hanna L.G., Harvey S.M., Moy L., DeMartini W.B., Dogan B. (2019). Utility of Diffusion-weighted Imaging to Decrease Unnecessary Biopsies Prompted by Breast MRI: A Trial of the ECOG-ACRIN Cancer Research Group (A6702). Clin. Cancer Res..

[16] Le Bihan D., Mangin J.F., Poupon C., Clark C.A., Pappata S., Molko N., Chabriat H. (2001). Diffusion tensor imaging: Concepts and applications. J. Magn. Reson. Imaging.

[17] Eyal E., Shapiro-Feinberg M., Furman-Haran E., Grobgeld D., Golan T., Itzchak Y., Catane R., Papa M., Degani H. (2012). Parametric diffusion tensor imaging of the breast. Investig. Radiol..

[18] Shapiro-Feinberg M., Weisenberg N., Zehavi T., Furman-Haran E., Grobgeld D., Nissan N., Degani H. (2012). Clinical results of DTI. Eur. J. Radiol..

[19] Furman-Haran E., Grobgeld D., Nissan N., Shapiro-Feinberg M., Degani H. (2016). Can diffusion tensor anisotropy indices assist in breast cancer detection?. J. Magn. Reson. Imaging.

[20] Nissan N., Furman-Haran E., Feinberg-Shapiro M., Grobgeld D., Eyal E., Zehavi T., Degani H. (2014). Tracking the mammary architectural features and detecting breast cancer with magnetic resonance diffusion tensor imaging. J. Vis. Exp..

[21] Scaranelo A.M., Degani H., Grobgeld D., Talbot N., Bodolai K., Furman-Haran E. (2020). Effect of IV Administration of a Gadolinium-Based Contrast Agent on Breast Diffusion-Tensor Imaging. AJR Am. J. Roentgenol..

[22] Baxter G.C., Graves M.J., Gilbert F.J., Patterson A.J. (2019). A Meta-analysis of the Diagnostic Performance of Diffusion MRI for Breast Lesion Characterization. Radiology.

[23] Wang K., Li Z., Wu Z., Zheng Y., Zeng S.E.L., Liang J. (2019). Diagnostic Performance of Diffusion Tensor Imaging for Characterizing Breast Tumors: A Comprehensive Meta-Analysis. Front Oncol..

[24] Iima M., Partridge S.C., Le Bihan D. (2020). Six DWI questions you always wanted to know but were afraid to ask: Clinical relevance for breast diffusion MRI. Eur. Radiol..

[25] Thomassin-Naggara I., Tardivon A., Chopier J. (2014). Standardized diagnosis and reporting of breast cancer. Diagn. Interv. Imaging.

[26] Berger N., Varga Z., Frauenfelder T., Boss A. (2017). MRI-guided breast vacuum biopsy: Localization of the lesion without contrast-agent application using diffusion-weighted imaging. Magn. Reson. Imaging.

[27] Montemezzi S., Cardano G., Storer S., Cardobi N., Cavedon C., Camera L. (2021). MRI-guided breast biopsy based on diffusion-weighted imaging: A feasibility study. Eur. Radiol..

[28] Jeong S., Kim T.H. (2022). Diffusion-weighted imaging of breast invasive lobular carcinoma: Comparison with invasive carcinoma of no special type using a histogram analysis. Quant. Imaging Med. Surg..

[29] Pinker K., Moy L., Sutton E.J., Mann R.M., Weber M., Thakur S.B., Jochelson M.S., Bago-Horvath Z., Morris E.A., Baltzer P.A. (2018). Diffusion-Weighted Imaging with Apparent Diffusion Coefficient Mapping for Breast Cancer Detection as a Stand-Alone Parameter: Comparison with Dynamic Contrast-Enhanced and Multiparametric Magnetic Resonance Imaging. Investig. Radiol..

[30] McKay J.A., Church A.L., Rubin N., Emory T.H., Hoven N.F., Kuehn-Hajder J.E., Nelson M.T., Ramanna S., Auerbach E.J., Moeller S. (2020). A Comparison of Methods for High-Spatial-Resolution Diffusion-weighted Imaging in Breast MRI. Radiology.

[31] Otikovs M., Nissan N., Furman-Haran E., Anaby D., Allweis T.M., Agassi R., Sklair-Levy M., Frydman L. (2021). Diffusivity in breast malignancies analyzed for b > 1000 s/mm^2^ at 1 mm in-plane resolutions: Insight from Gaussian and non-Gaussian behaviors. J. Magn. Reson. Imaging.

